# PD-L1 expression and its relationship with oncogenic drivers in non-small cell lung cancer (NSCLC)

**DOI:** 10.18632/oncotarget.15839

**Published:** 2017-03-01

**Authors:** Liyan Jiang, Xinying Su, Tianwei Zhang, Xiaolu Yin, Meizhuo Zhang, Haihua Fu, Hulin Han, Yun Sun, Lili Dong, Jialin Qian, Yanhua Xu, Xuan Fu, Paul R. Gavine, Yanbin Zhou, Kun Tian, Jiaqi Huang, Dong Shen, Haiyi Jiang, Yihong Yao, Baohui Han, Yi Gu

**Affiliations:** ^1^ Shanghai Chest Hospital, Shanghai Jiao Tong University, Shanghai, China; ^2^ Asia & Emerging Markets, iMed, AstraZeneca, Shanghai, China; ^3^ R&D Information, AstraZeneca, Shanghai, China; ^4^ Global Medicines Development, AstraZeneca, Shanghai, China; ^5^ The First Affiliated Hospital, Sun Yat-Sen University, Guangdong, China; ^6^ General Hospital of Chengdu Military Region of PLA, Sichuan, China; ^7^ R&D, MedImmune, AstraZeneca, Gaithersburg, MD, USA

**Keywords:** PD-L1, non-small cell lung cancer (NSCLC), biomarker, tumor-infiltrating immune cells, oncogenic driver

## Abstract

In order to explore the potential patient population who could benefit from anti PD-1/PD-L1 mono or combination therapies, this study aimed to profile a panel of immunotherapy related biomarkers (PD-1, PD-L1, CTLA-4 and CD8) and targeted therapy biomarkers (EGFR, KRAS, ALK, ROS1 and MET) in NSCLC.

Tumor samples from 297 NSCLC patients, including 156 adenocarcinomas (AD) and 129 squamous cell carcinomas (SCC), were analyzed using immunohistochemistry, immunofluorescence, sequencing and fluorescence *in situ* hybridization.

43.1% of NSCLC patients had PD-L1 positive staining on ≥ 5% tumor cells (TC). Furthermore, dual color immunofluorescence revealed that the majority of PD-L1/CD8 dual positive tumor infiltrating lymphocytes (TIL) had infiltrated into the tumor core. Finally, combined analysis of all eight biomarkers showed that tumor PD-L1 positivity overlapped with known alterations in NSCLC oncogenic tumor drivers in 26% of SCC and 76% of AD samples.

Our illustration of the eight biomarkers’ overlap provides an intuitive overview of NSCLC for personalized therapeutic strategies using anti-PD-1/PD-L1 immune therapies, either as single agents, or in combination with targeted therapies. For the first time, we also report that PD-L1 and CD8 dual positive TILs are predominantly located within the tumor core.

## INTRODUCTION

Lung cancer is the most common cause of cancer related mortality, estimated to be responsible for 1.59 million deaths, representing 19.4% of all cancer deaths worldwide in 2012 (GLOBOCAN2012). Non-small cell lung cancer (NSCLC), accounting for about 85% of all lung cancer, compromises different histological subtypes which include adenocarcinoma (AD), squamous cell carcinoma (SCC), large cell carcinoma and other types. Despite advances in improving early diagnosis, combining surgery with chemotherapy and/or radiotherapy, and understanding molecular genetics to apply personalized therapies, NSCLC still has a poor prognosis with 5-year relative survival rates remaining around 15% [1]. New therapeutic strategies therefore, are urgently required.

Cancer immunotherapy was crowned as ‘Breakthrough of the Year 2013’ in celebrating its remarkable efficacy in many cancer patients [2]. Under normal conditions, immune checkpoints are involved in balancing pro- and anti-immune reactions [3]. Within tumor tissues, however, tumor cells (TC) hijack immuno-inhibitory mechanisms to facilitate evasion of the immune system's attack, thus ensuring tumor survival [4]. Blockade of immune checkpoints, such as cytotoxic T-lymphocyte antigen-4 (CTLA-4) and programmed cell death-1 (PD-1)/ programmed cell death ligand-1 (PD-L1) signaling, have demonstrated broad anti-cancer activities with an acceptable side effect profile in different cancer types, including NSCLC [5–9].

PD-L1 is the major ligand of PD-1 and shows normal broad expression in dendritic cells, macrophages, mast cells, T cells, B cells, endothelial and epithelial cells [10]. Within the tumor environment, PD-L1 expression is ectopically up-regulated on TC as well as on tumor infiltrating immune cells (IC) [8]. Several clinical trials in NSCLC have demonstrated a correlation between increased PD-L1 expression on NSCLC TC and/or tumor infiltrating IC with enhanced efficacy of anti- PD-1/PD-L1 immunotherapies [7, 8, 11–13]. Keytruda (Pembrolizumab) was approved by the FDA as second line treatment for PD-L1 positive NSCLC patients. Opdivo (Nivolumab) also obtained FDA approval for use in second line NSCLC, regardless of PD-L1 expression status. Therefore, further analysis of PD-L1 expression is warranted in order to evaluate the potential of PD-L1 as a patient selection biomarker to enrich for patients who could ultimately benefit from anti-PD-1/PD-L1 therapies.

Following demonstration of monotherapy efficacy, a number of NSCLC clinical trials are now exploring anti-PD-1/PD-L1 antibodies in combination with chemotherapy [14, 15] or with molecularly targeted therapies [16, 17]. The phase I ‘CheckMate-012’ study which combined Nivolumab with platinum-based doublet chemotherapy, demonstrated hints of synergistic effects in several NSCLC patients [18], whilst in a separate study, the combination of Atezolizumab with nab-paclitaxel plus carboplatin showed more promising synergy [15]. Very recently, the KEYNOTE-021 study demonstrated clinical efficacy using Pembrolizumab plus chemotherapy in treatment-naive advanced NSCLC, regardless of Pembrolizumab dose or PD-L1 status [15]. Combination of two immune checkpoint antagonists has shown more promising synergistic efficacy. A phase I study combining Durvalumab (MEDI4736) with Tremelimumab demonstrated improved tumor responses in advanced NSCLC patients, compared to single agent therapy alone, regardless of PD-L1 status [19]. With regard to TKI drug combinations, in a 21 patient phase I study of Erlotinib plus Nivolumab in EGFR mutant NSCLC, promising efficacy was observed with a PFS of 29.4 weeks and an ORR of 19% [20]. Given the potential advantages of strategies combining immune checkpoint inhibitors and TKIs, it is important to understand any correlations between immune mediated therapy for cancer (IMT-C) related biomarkers and NSCLC tumor drivers.

Thus, in order to assess the population of patients who could potentially benefit from single agent or combination therapies using immune checkpoint inhibitors, we profiled a panel of IMT-C related biomarkers (PD-1, PD-L1, CTLA-4 and CD8) and oncogenic biomarkers with relevant targeted therapeutic drugs (*EGFR*, *KRA*S, *ALK*, *ROS1* and *MET*) in NSCLC.

## RESULTS

### Expression of PD-L1 on tumor cells (TC) and its relationship with lung cancer driver genes

**Figure 1:**
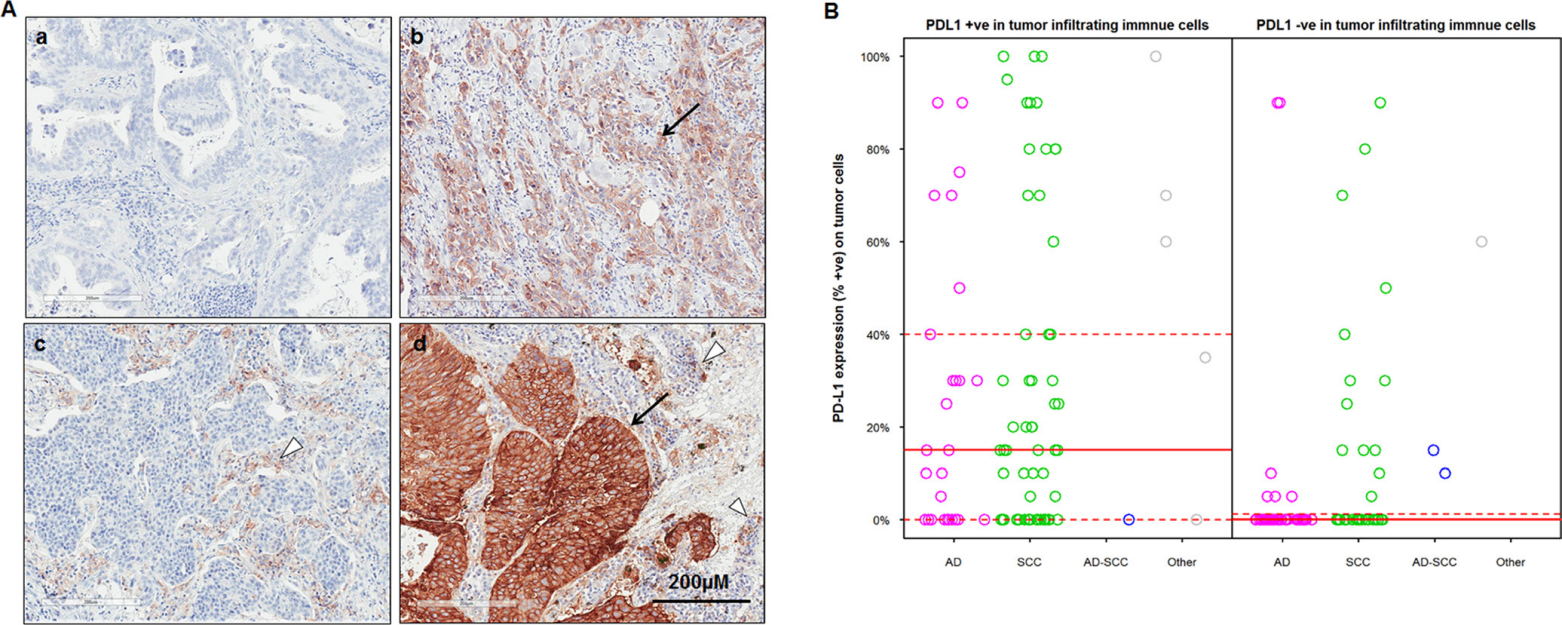
PD-L1 expression on tumor cells (TC) and tumor infiltrating immune cells (IC) in Chinese NSCLC patients (**A**) representative images on adenocarcinomas (a & b) and squamous carcinomas (c &d) showed PD-L1 negative staining (a), positive staining on TC (b), IC (c) and both on TC and IC (d) respectively by IHC. The arrows indicate PD-L1 positivity on TC, the triangle indicates PD-L1 positivity on IC; (**B**) Correlation between PD-L1 expression on TC and PD-L1 positivity in IC in 182 patients. Median (solid red lines) and the 1^st^ and 3^rd^ quantile (dashed red lines) are reported.

PD-L1 expression on tumor cells was successfully evaluated on 297 Chinese NSCLC patient samples including 156 AD, 129 SCC and 12 other types. The patients’ clinical information are summarized in Table 1. PD-L1 expression of any intensity was observed in 128 (43.1%) cases, with positive staining in at least 5% TC. 73 (24.6%) cases had positive staining in ≥25% TC. 45 (15.2%) cases had positive staining in ≥50% TC (Figure 1 and Table 2). The PD-L1 positive prevalence on TC was significantly higher in SCC than in AD when using ≥5% as a cut off (49.6% vs 36.5%, *p* = 0.0304) or marginally significantly higher when using ≥ 25% (29.5% vs. 19.2%, *p* = 0.0509) as a cut off.

**Table 1:** Patients’ clinical information

Clinical Characteristics	All	AD	SCC	AD-SCC	Others
**Gender**	**297**	**156**	**129**	**4**	**8**
Female	86	81	3	1	1
Male	211	75	126	3	7
**Age**	**297**	**156**	**129**	**4**	**8**
< 65	161	102	52	1	6
≥ 65	136	54	77	3	2
**Smoking Status**	**297**	**156**	**129**	**4**	**8**
Ever smoker	169	55	104	3	7
Never smoker	128	101	25	1	1
**Tumor Grade**	**297**	**156**	**129**	**4**	**8**
1	14	7	6	0	1
2	168	86	79	2	1
3	111	60	43	2	6
4	4	3	1	0	0
**Clinical Stage**	**297**	**156**	**129**	**4**	**8**
I-IIIa (resectable)	206	94	101	4	7
IIIb-IV (non-resectable)	91	62	28	0	1
**T**	**297**	**156**	**129**	**4**	**8**
1	22	16	6	0	0
2	164	82	77	0	2
3	61	26	28	3	6
4	50	32	18	1	0
**N**	**297**	**156**	**129**	**4**	**8**
0	83	53	28	1	1
1	72	27	40	1	4
2	110	54	52	2	2
3	32	22	6	0	1
**M**	**297**	**156**	**129**	**4**	**8**
0	224	100	113	4	7
1	73	56	16	0	1

**Table 2:** Biomarkers’ characterization on 297 NSCLC patients

Biomarkers	*n* (%) in all	*n* (%) in SCC	*n* (%) in AD
**PD-L1 expression on TC**	**297**	**129**	**156**
≥ 5%	128 (43.1%)	64 (49.6%)	57 (36.5%)
≥ 25%	73 (24.6%)	38 (29.5%)	30 (19.2%)
≥ 50%	45 (15.2%)	21 (16.3%)	20 (12.8%)
**PD-L1 expression on IC**	**183**	**95**	**79**
> 1%	95 (51.9%)	65 (65.3%)	27 (34.2%)
> 10%	53 (29.0%)	30 (31.6%)	19 (24.1%)
**PD-1 expression on IC**	**190**	**96**	**85**
+ve	169 (88.9%)	84 (87.5%)	78 (91.8%)
−ve	21 (11.1%)	12 (12.5%)	7 (8.2%)
**CTLA 4 expression on IC**	**190**	**101**	**80**
+ve	185 (97.4%)	99 (98.0%)	77 (97.5%)
−ve	5 (2.6%)	2 (2.0%)	3 (2.5%)
**EGFR Mutation on TC**	**297**	**129**	**156**
+ve	75 (25.3%)	10 (7.8%)	63 (40.4%)
−ve	222 (74.7%)	119 (92.2%)	93 (59.6%)
**KRAS Mutation on TC**	**240**	**107**	**124**
+ve	14 (5.8%)	2 (1.9%)	10 (8.1%)
-ve	226 (94.2%)	105 (98.1%)	114 (91.9%)
**MET expression on TC (IHC 2/3+ ≥75%)**	**291**	**126**	**153**
+ve	35 (12.0%)	9 (7.1%)	23 (15.0%)
-ve	256(88.0%)	117 (92.9%)	130 (85.0%)
**MET gene copy number on TC(≥5 copies)**	**183**	**79**	**96**
+ve	5 (2.7%)	0 (0.0%)	5 (5.2%)
-ve	178 (97.3%)	79 (100.0%)	91 (94.8%)
**ALK rearrangements on TC**	**294**	**128**	**154**
+ve	12 (4.1%)	3 (2.3%)	9 (5.8%)
-ve	282 (94.9%)	125 (97.6%)	145 (94.2%)
**ROS1 rearrangements on TC**	**255**	**115**	**130**
+ve	3 (1.20%)	0 (0.0%)	3 (2.3%)
-ve	252 (98.8%)	115 (100.0%)	127 (97.7%)

TC: tumor cells; IC: tumor infiltrating immune cells; IHC: immunohistochemistry

**Figure 2:**
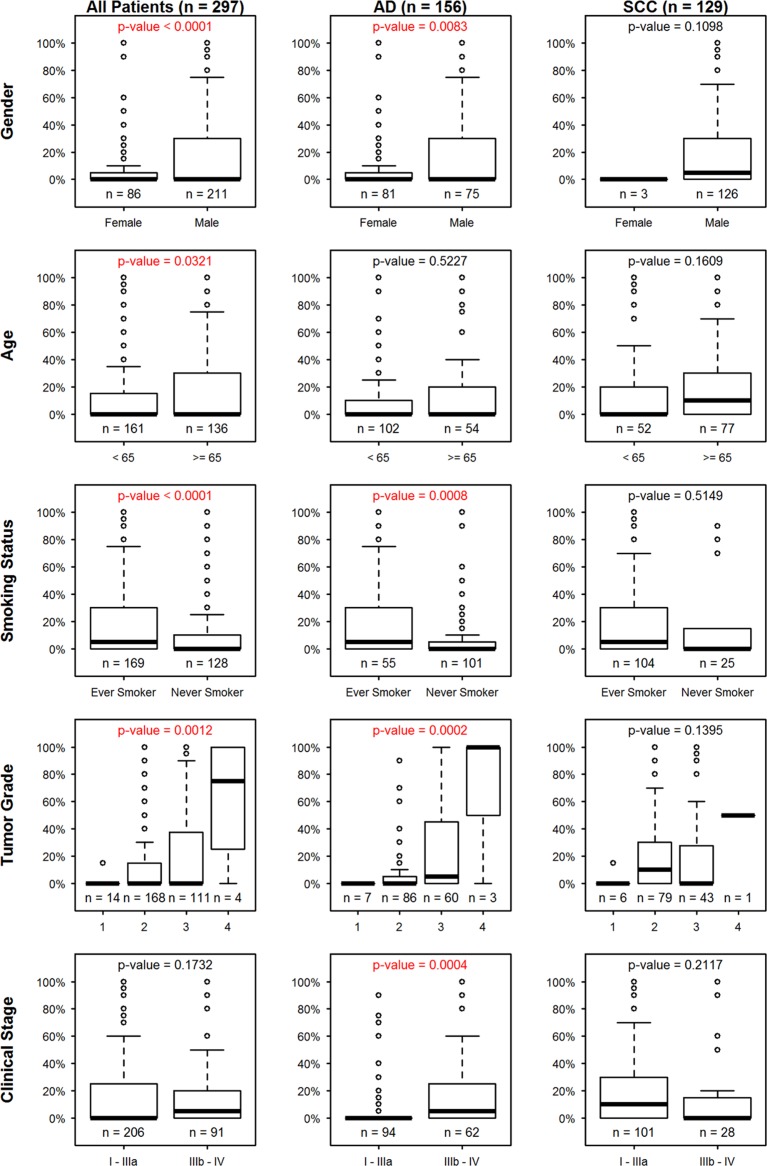
Association between PD-L1 expression (%+ve) on tumor cells (TC) and clinical parameters in the whole patient cohort, adenocarcinoma (AD) and squamous cell carcinoma (SCC) subgroups in the first, second and third column, respectively Each row signifies a clinical parameter.

The correlation between tumor cell PD-L1 expression and patients’ clinical parameters was analyzed within the whole cohort. Patients’ histologic grade and clinical stages were defined according to the 7^th^ edition of the AJCC staging system based on tumor size and extension (T), lymph node involvement (N) and the presence of distant metastasis (M) [21]. PD-L1 expression on TC was higher in male (*p* < 0.0001), older (*p* = 0.0321), smoker (*p* < 0.0001), high histologic grade (*p* = 0.0012) or SCC (*p* = 0.0412) patients, but did not show any difference when comparing surgically resectable (stage I-IIIa) with non-resectable (stage IIIb-IV) patients (Figure 2). The associations between PD-L1 expression on TC and clinical parameters were further analyzed in detail by separating SCC and AD patients into two groups. Interestingly, no statistical significance was observed with any clinical parameter within the SCC group, suggesting that the statistical difference came mainly from the AD group. In the AD subgroup, PD-L1 expression on TC was significantly higher in male (*p* = 0.0083), smoker (*p* = 0.0008), higher histologic grade (*p* = 0.0002) and surgically non-resectable (*p* = 0.0004) patients. The higher grades of regional lymph node metastatic (*p* = 0.0064) and distant metastatic (*p* = 0.0058) patient tumors contributed to the significance of higher PD-L1 expression in later stage patients.

**Figure 3:**
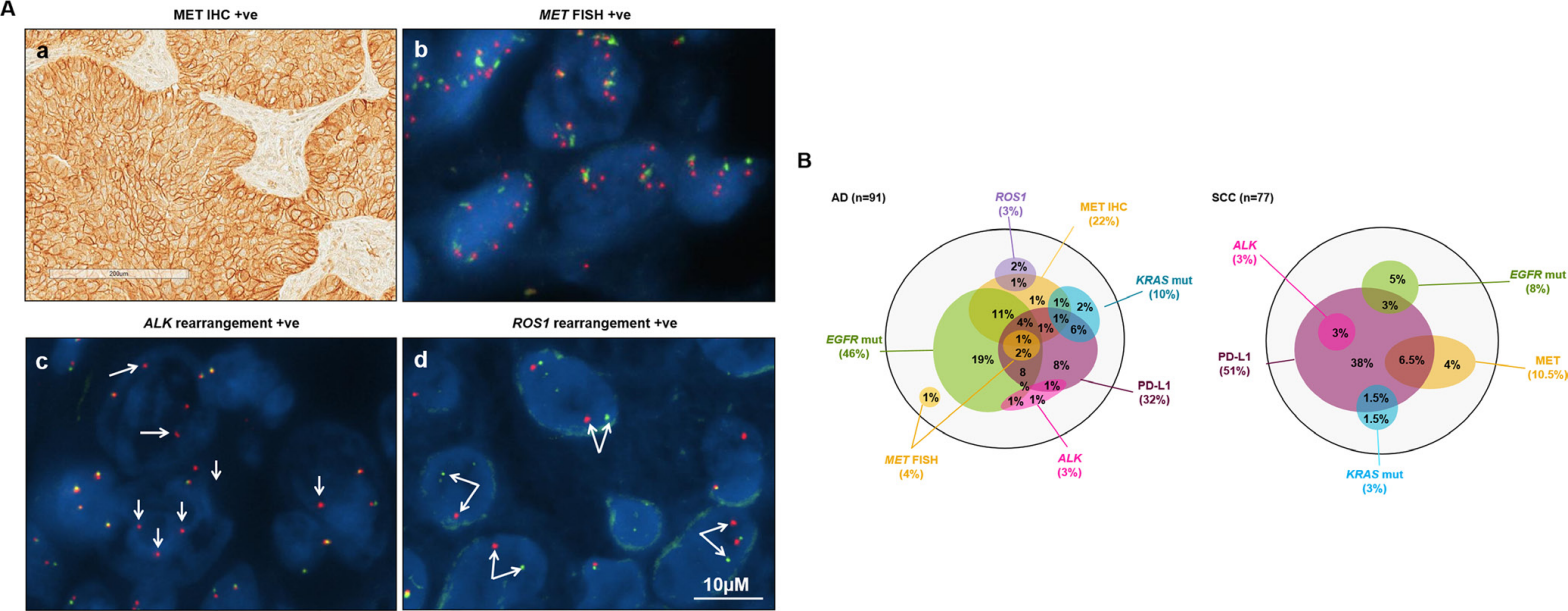
Association between PD-L1 positive staining on tumor cells (TC) and other biomarkers of lung cancer drivers (**A**) representative images of lung cancer drivers including (a) MET IHC positive, (b) *MET* FISH positive, (c) *ALK* and (d) *ROS1* rearrangement positive. For FISH images, the red signals represent C-termini of *ALK* or *ROS1* genes, the green signals represent N-termini of *ALK* or *ROS1* genes, nucleus of tumor cells were stained as blue by DAPI; (**B**) the relationship of PD-L1 on TC and lung cancer driver genes in AD and SCC patients respectively. The largest black circles represent 91 AD and 77 SCC patients respectively. The size of each circle reflects the patient number.

To understand the relationship between tumor cell PD-L1 positivity and other lung cancer tumor driver gene alterations, especially those with available targeted therapeutic agents, we profiled *EGFR, KRAS, MET, ALK* and *ROS1* gene abnormalities in the same cohort, dependent on tumor sample availability (Table 2 and Figure 3). *EGFR* mutations were detected in 75 out of 297 cases (25.3%) including 10 SCC and 63 AD. There were two *EGFR* resistance mutations observed in the AD group, one was T790M/exon19 deletion dual mutation and the other was exon 20 insertion. Both patients were PD-L1 negative. *KRAS* mutations were found in 14 out of 240 cases (5.8%) including 2 SCC and 10 AD patients. Among 5 (out of 183) patients with more than 5 copies of the *MET* gene, all were AD but none were SCC. MET protein expression positivity was observed in 35 out of 291 (12.0%), occurring in 9 SCC and 23 AD patients (3 other types). *ALK* rearrangements were observed in 12 patient samples (out of 294, 4.1%) including 3 SCC and 9 AD. Three *ROS1* rearranged patients were identified only within the AD group. None of the five aforementioned NSCLC driver gene aberrations showed any statistical association with PD-L1 positivity.

Overall, there were 91 AD and 77 SCC cases with completed analysis of tumor cell PD-L1 expression and all 5 NSCLC driver genes. Within the AD group, there were 42 (46%) *EGFR* mutated and 3 (3%) *ALK* rearranged cases, including one *EGFR/ALK* dual positive case. Approximately one third (15/44) of these *EGFR/ALK* tyrosine kinase inhibitor (TKI)-eligible patients had PD-L1 expression on ≥5% of TC. Two thirds (6/9) of *KRAS* mutant patient samples and three quarters (3/4) of *MET* FISH positive samples also had PD-L1 expression on ≥5% of TC. Within the SCC group, there were 6 *EGFR* mutated and 2 *ALK* rearranged cases. 4 out of 8 *EGFR/ALK* TKI-eligible patients and 1 out of 2 *KRAS* mutant patient samples had PD-L1 expression on ≥5% of TC. When using a PD-L1 positivity cut-off of ≥5% of tumor cells, there were 76% (22 out of 29) PD-L1 positive AD patient samples which harbored one or more of the 5 LC tumor driver alterations. Since the PD-L1 positivity rate in SCC was higher and LC tumor driver abnormalities were rarer compared to AD, only 26% (10 out of 39) of PD-L1 positive SCC patient samples harbored one or more of the LC tumor driver gene abnormalities. When using PD-L1 positivity on ≥ 50% of tumor cells as a cut off, despite the decrease in positive case numbers, the percentage of overlapping cases remained similar, with 67% AD (8 out of 12) and 33% SCC (4 out of 12) cases showing overlap.

### Expression of PD-L1, PD-1 and CTLA-4 on tumor infiltrating immune cells (IC)

Expression of PD-L1 on tumor infiltrating immune cells (IC) was observed in 52% of patient samples (95 out of 183), including 23% (42/183) cases showing PD-L1 expressed on 1-10% of IC and 29% (53/183) showing PD-L1 expressed on >10% of IC (Figure 1A and Table 2). Of these 183 cases, the majority were surgical samples with only 18 cases representing biopsies. Again, SCC patients had a higher prevalence of PD-L1 expression on IC when compared to AD patients when using >1% as cut off (65.3% vs 34.2%, *p* < 0.0001) (Table 2). When comparing PD-L1 expression on TC and tumor infiltrating IC among these 183 patients, 62 cases had PD-L1 positive expression on both TC and IC, and 66 cases were PD-L1 negative on either TC or IC. In total, approximately 70% of cases had the same PD-L1 staining status on both TC and IC. This correlation was statistically significant (*p* < 0.0001) (Figure 1B).

The expression of PD-1 and CTLA-4 on tumor infiltrating IC was successfully evaluated in 190 patients. Almost all patients showed PD-1 (88.9%; 169/190) or CTLA-4 (97.4%; 185/190) positivity on IC. The positive staining prevalence of both biomarkers was comparable between SCC and AD (Table 2). Furthermore, among PD-L1 TC positive patients, 73 cases (out of 83, 88.0%) also harbored PD-1 positive staining on IC and 82 cases (out of 85, 96.5%) were CTLA-4 positive on IC.

### Interactions between tumor cells (TC) and tumor infiltrating immune cells (IC)

**Figure 4:**
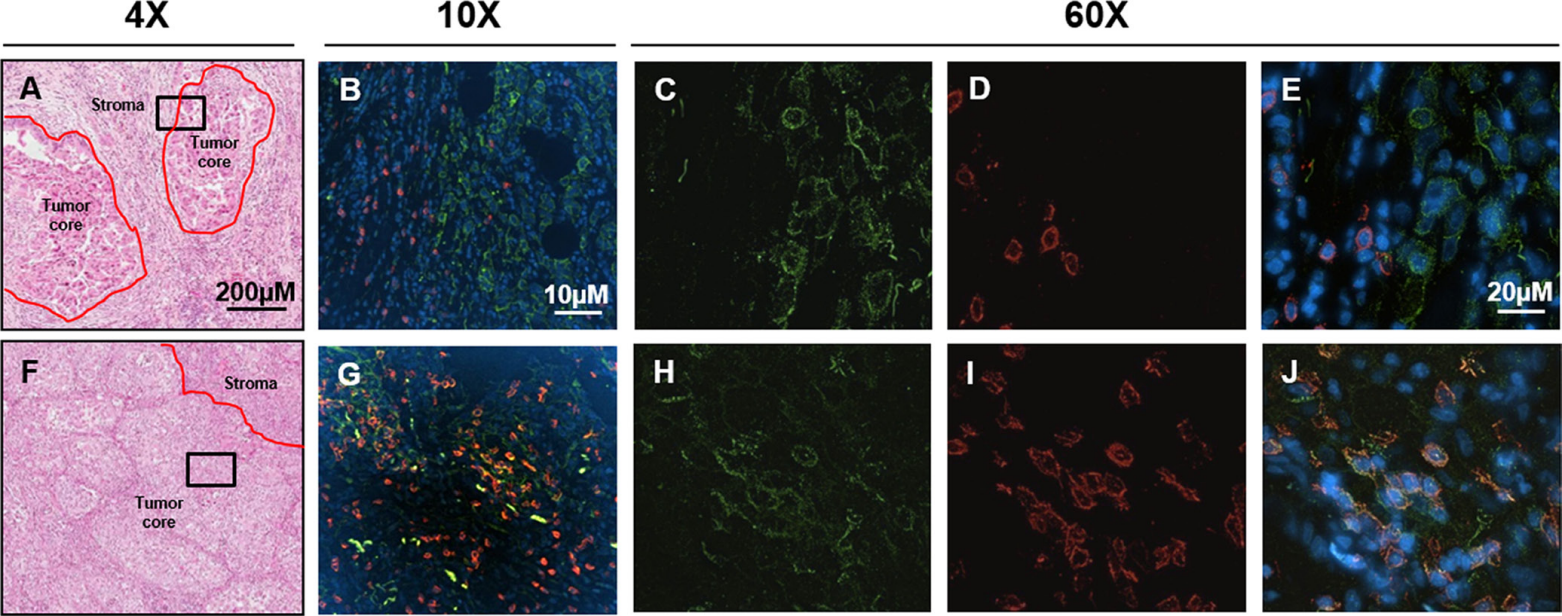
Location of CD8+ lymphocytes in Chinese NSCLC tumors (**A** and **F**) are representative images showing CD8+ lymphocytes locating in the stroma and tumor center respectively. The black square indicates the same tissue location for fluorescent images. (**B**–**E**) showed that at stroma CD8 and PD-L1 were not co-localized, while (**G**–**J**) showed colocalization of CD8 and PD-L1 on lymphocytes which mainly occurred in the tumor center (as indicated by arrows). PD-L1 was stained as green, CD8 was stained as red and the nucleus was counterstained as blue by DAPI.

In order to explore interactions between the tumor and immune system, PD-L1 and CD8 expression on tumor cells and/or immune cells was assessed using a dual immunofluorescence (IF) assay on surgically resected samples from 95 NSCLC patients. CD8 positive tumor infiltrating lymphocytes (TILs) presented in all 95 cases. Based on the tumor cell PD-L1 staining status, we classified these 95 NSCLC patient samples into two groups: PD-L1 positive (45 samples, 47%) and PD-L1 negative (50 samples, 53%). The positive group was represented by a higher number of SCC patients (25/45), whilst AD patients were dominant in the negative group (32/50). Furthermore, we found that CD8 positive TILs localized to different tumor areas between the two groups. 71% (32/45) of PD-L1 positive group tumors had CD8+ lymphocytes infiltrated within the tumor core, while 98% (49/50) of PD-L1 negative group tumors had CD8+ lymphocytes distributed only within the stroma (Figure 4A).

Finally, PD-L1 and CD8 dual positive lymphocytes were identified in 34 patient samples. Interestingly, 27 (79%) of them had PD-L1/CD8 dual stained lymphocytes infiltrated within the tumor core (Figure 4J). Furthermore, 26/27 (96%) PD-L1/CD8 dual positive lymphocytes were only observed in the tumor core.

## DISCUSSION

Given that evidence of significant and durable anti-tumor efficacy has been demonstrated using PD-1/PD-L1 inhibitors in several NSCLC clinical trials [5–9], numerous efforts are ongoing to identify determinants of response, including PD-L1 expression [5–9, 22, 23], CD8+ TIL [24], smoking status [7–9] and mutation/neoantigen burden [25].

Due to some tantalizing hints of correlations between clinical responses and high PD-L1 expression on TC and/or tumor infiltrating IC [7, 8, 11–13], many research groups have opted to study PD-L1 expression on clinical or preclinical NSCLC samples. Using immunohistochemistry (IHC) assays, several Chinese teams have reported prevalences of tumor cell PD-L1 cytoplasmic and/or membrane staining ranging from 39.9% to 65.9% on Chinese NSCLC patient samples [26–31]. In our study, only PD-L1 membrane staining was used to define PD-L1 positivity, generating a positive staining prevalence of 43.1%, which is largely consistent with the 39.9% reported by Yang's group [29]. With regard to correlations between clinicopathological parameters and NSCLC tumor cell PD-L1 expression, inconsistent data have been reported by different research groups [26, 28, 29, 32–36], which we speculate could be attributable to differences in cohort size, unbalanced enrollment of histopathological subtypes and differing IHC assays or data interpretation methodologies. Accordingly, a more detailed analysis of PD-L1 expression in a cohort with an evenly distributed AD/SCC profile and early/late stage patient population was important. To this end, we collected 156 AD and 129 SCC, including 206 surgically resectable and 91 surgically non-resectable patient samples. In this study, we found that tumor cell PD-L1 expression was significantly correlated with age, gender, smoking status, histologic grade and histopathological subtype. The higher tumor cell PD-L1 expression in male, smoker, SCC and higher histologic grade samples was consistent with data from a Korean study comprising a large cohort of 779 Korean NSCLC [37]. Since male gender, smoking history and SCC are three factors which are typically extremely closely correlated with each other [38], we were not able to distinguish them for multivariate analysis and therefore it was difficult to precisely determine which factor(s) had a dominant contribution to the statistical significances we observed. When we did further analysis by separating AD and SCC into two groups, the statistical significances shown in whole cohort remained in AD, but not in SCC, suggesting that the PD-L1 expression status differs between NSCLC subtypes. Our data show that in AD, higher PD-L1 expression is enriched in more advanced disease samples (higher histologic grade or later stage), whilst in SCC a higher PD-L1 expression prevalence correlates only with this subtype, but not with the individual patient disease stage.

The correlation between clinical response to PD-1/PD-L1 inhibitors and increased tumor cell PD-L1 on TC or tumor infiltrating IC has been reported previously. The FDA approved both the “narrow” usage of Keytruda in PD-L1 positive NSCLC and “broad” usage of Opdivo as second line treatments, regardless of PD-L1 expression status. Whether or not to use PD-L1 expression as a prospective patient selection biomarker for anti-PD-1/PD-L1 immunotherapies remains a challenging question. In this study, the anti-PD-L1 antibody E1L3N was used for IHC analysis. This antibody generated comparable data to that of the SP263 antibody used in AstraZeneca's clinical trials (internal unpublished data). With E1L3N, we have found that the prevalence of PD-L1 positivity on both TC and IC were higher in SCC than in AD. Since all samples used for this study were from treatment naive NSCLC patients, we were not be able to provide a PD-L1 positivity cut off to correlate with clinical response to immunotherapy. In all statistical analyses, PD-L1 expression on tumor cells was treated as a continuous variable instead of using a positivity cut off. Furthermore, the IC were usually diffused in the tumor centers or stroma, which made it challenging for the pathologists to provide a precise percentage. Therefore, our IHC positivity criteria for biomarkers (PD-L1, PD-1, OX40 and CTLA-4) on IC was defined as any positive staining on IC, regardless of percentage.

In the Keynote 001 study, 10.7% of NSCLC patients (3/28) with PD-L1 staining of < 1% achieved an overall response upon Pembrolizumab treatment [7]. This data clearly indicates that other factors are relevant in determining the patient response to immunotherapy. The presence of CD8+ lymphocytes within the tumor core or microenvironment is certainly of interest.

To explore the incidence of CD8+ lymphocytes and their colocation within NSCLC tumors, dual color IF for PD-L1 and CD8 was used to analyze 95 NSCLC patient samples in our study. Our PD-L1 positive and negative groups matched perfectly with the type I and type IV tumors respectively proposed by Teng [39]. According to Teng *et al*, Type I patient tumors which were PD-L1 positive and contained TILs were most likely to respond to PD-1/PD-L1 inhibitors. Interestingly, our data show that within the PD-L1 positive group, there is a significant enrichment of SCC tumors *versus* AD. The majority of the PD-L1 tumor positive group patient samples contained CD8+ lymphocytes infiltrated into the tumor core, whilst the PD-L1 negative group patient samples displayed CD8+ lymphocytes largely within the stroma. Furthermore, PD-L1 and CD8 dual positive lymphocytes were largely observed to have infiltrated within the centers of tumors with high PD-L1 expression. Based on our data and known mechanisms, these observations suggest that tumor core colocalization of PD-L1 positive tumor cells and PD-L1/CD8 dual positive lymphocytes may represent the optimal scenario for maximal tumor cell killing when treating with PD-1/PD-L1 inhibitors.

Even within PD-L1 positive NSCLC patient subgroups, the best objective response rate using single agent anti-PD-1/PD-L1 therapies has been around 45%. In order to enhance and extend the therapeutic benefit of immunotherapy to a broader population, a variety of combination strategies have been proposed or tested clinically. In this study, we explored the overlap between PD-L1 tumor cell expression and genetic alterations in commonly known oncogenic driver genes associated with LC. Our data reveal that the degree of overlap is much higher in AD than in SCC (76% vs 26%) and that furthermore, almost all patients with significant overlap expressed PD-1 or CTLA-4 on IC. The overlap between PD-L1 TC positive and PD-1 or CTLA-4 positive on IC were both very high, at 88.0% and 96.5%, respectively. Fully understanding the relationships between PD-L1 expression and other immune checkpoints, as well as response biomarkers for existing targeted therapies, will be crucial in guiding the development of rational combination approaches.

Our biomarker data also showed interesting differences between SCC and AD. Overall, a higher proportion of SCC samples were PD-L1 positive, regardless of the patients’ clinical characteristics. These patient tumors also had higher CD8+ lymphocyte counts, especially within the tumor core and showed less PD-L1/oncogenic driver overlap than AD. In contrast, AD had a lower proportion of PD-L1 positive patients. The PD-L1 positive population was enriched in older, smoker and more advanced stage patients and two thirds of the PD-L1 positive AD tumor samples showed overlap with LC genetic drivers. Accordingly, these different disease characteristics suggest that SCC and AD patients may potentially require different therapeutic strategies to derive maximal benefit. For instance, SCC patients may ultimately be more responsive to immunotherapy while AD patients may potentially benefit more from combination approaches utilizing IMT-C with TKIs.

In conclusion, this is the first comprehensive study of a large NSCLC cohort, not only to explore the expression of PD-L1 on tumor and infiltrating immune cells, but also to analyze the relationship between PD-L1 expression and other important NSCLC genetic drivers. Also, for the first time, we report that PD-L1 and CD8 dual positive TILs are predominantly located within the tumor cores. Taken together, these results suggest that there may be potential in using therapeutic strategies combining anti PD-1/PD-L1 immune therapies as either single agents or in combination with targeted therapies.

## MATERIALS AND METHODS

### Patients and tumor samples

NSCLC tumor specimens from 297 patients were collected and included 214 cases from the Shanghai Chest Hospital (Shanghai, China) collected between 2009 and 2012, and 83 cases from the ‘IGNITE’ clinical trial (D7913C00074). Prior written informed consent was obtained from all patients and the study protocol was approved by the ethics committees at Shanghai Chest hospital. These samples comprised 235 surgical samples and 62 biopsies. Formalin fixed and paraffin embedded (FFPE) sections were stained with hematoxylin and eosin (H&E) and reviewed by pathologist to confirm the NSCLC diagnosis for all cases.

To achieve a more objective statistical comparison between AD and SCC, 156 AD and 129 SCC were collected. For the same reason, 91 surgically non-resectable (stage IIIb-IV) cases were enrolled in order to more accurately compare with the 206 surgically resectable (stage I-IIIa) cases. Patients’ median age was 65 years and 169 patients were ‘ever smokers’ who had current or previous smoking histories (Table 1).

### Immunohistochemistry (IHC)

IHC assays were used to detect the expression of PD-L1, PD-1, CTLA-4 and MET on FFPE tissue sections. For PD-L1 IHC, placenta and MDA-MB-231 were used as positive control whilst MCF-7 was used as negative control. For PD-1, CTLA-4 and OX40 IHC, tonsil was used as positive control. GC cell lines with different MET expression levels were used as control for MET IHC.

PD-L1 and PD-1 IHC staining were performed using a rabbit anti-human PD-L1 (E1L3N) monoclonal antibody (1:300, CST#13684, Cell Signaling Technology) or anti-PD-1 antibody (1:30, HPA035981, Atlas antibodies). The IHC procedure is described briefly as follows. Deparaffinized and rehydrated FFPE sections were immersed in high pH target retrieval solution in a pressure cooker (PTlink module, DAKO) at 97°C for either 35 minutes (PD-L1 staining) or 15 minutes (PD-1 staining). Peroxidase blocking was then performed as follows. For PD-L1, sections were treated with 2.5% H_2_O_2_ in methanol for 15 minutes, followed by an incubation of protein block solution (PBS with 2% cold water fish skin gelatin, 1% casein, 2% normal goat serum and 0.1% Tween-20) for 30 minutes. For PD-1, sections were treated with peroxidase blocking solution (S2023, DAKO) for 5 minutes. Sections were then incubated with primary antibody at room temperature for 60 minutes. After washing twice with TBS-T, sections were incubated with the EnVision+ -HRP labeled secondary antibody (K4003, DAKO) for 30 minutes. After a further two washes in TBS-T, slides were finally visualized using DAB substrate-chromagen (K3468, DAKO).

MET and CTLA-4 IHC staining was performed using a rabbit monoclonal anti-total cMET (SP44) antibody (790-4430, Ventana Medical Systems) and a goat anti-CTLA-4 monoclonal antibody (2.5ug/ml, AF-386-PB, R&D systems) using a Ventana automatic immunostainer (Discovery XT/Ultra; Ventana Medical Systems). MET staining followed the standard Ventana protocol, whilst the CTLA-4 staining procedure was as follows: antigen retrieval at 95°C for 64 minutes, primary antibody incubation at 37°C for 28 minutes and secondary antibody incubation at 37°C for 16 minutes.

For IHC result interpretation, PD-L1 expression was evaluated both on tumor cells (TC) and tumor infiltrating immune cells (IC), while PD-1, CTLA-4 and OX40 expression was only evaluated on IC. PD-L1 positivity on TC was defined by positive TC percentage regardless of staining intensity using 5%, 25% or 50% as cut off, while the positivity on IC was defined by any positive staining on IC using 1% or 10% as cut off. PD-1, CTLA-4 and OX40 positivity were defined the same as PD-L1 without cut off setting. The existence of IC was defined as the presence of immune cells located within the stroma or tumor core [40]. Necrotic areas and acute inflammatory cells were ignored for IC analysis. Positive MET expression was defined as IHC 2+ or 3+ (0 – 3+ scale) in ≥ 75% of tumor cells.

### DNA extraction

DNA was isolated from frozen tissues using the Puregene DNA extraction kit (Qiagen, Maryland, USA) according to the manufacturer's instructions. Extracted DNA was quantified using a Nanodrop 2000 Spectrophotometer (Thermo Fisher, USA). For EGFR mutation detection, the concentration of each DNA sample was normalized to 0.4 ng/μL. For KRAS mutation detection, the concentration of each DNA sample was normalized to 0.66 ng/μL.

### *EGFR* and *KRAS* mutation detection by amplification refractory mutation system (ARMS)

Human *EGFR* Gene 29 Mutations Fluorescence Polymerase Chain Reaction (PCR) Diagnostic Kit and Human *KRAS* Gene 7 Mutations Fluorescence Polymerase Chain Reaction (PCR) Diagnostic Kits (Amoy Diagnostics, Xiamen, China) were used for the *EGFR* and *KRAS* mutation detections in this study. All experiments were performed according to the manufacturer's instructions.

### Fluorescence *in situ* hybridization (FISH)

Dual-color FISH was performed to assess *MET* gene copy number change or *ALK* & *ROS1* gene rearrangements. The *MET* probe was generated by directly labeling BAC (CTD-2270N20) with SpectrumRed-dUTP (02N34-050, ENZO), and the CEP7- Spectrum Green probe (32-132007, Vysis) for the centromeric regions of chromosome 7 used as internal controls. The *ALK* break-apart FISH probe kit (06N43-020, Vysis) was used according to the manufacturer's instructions. The break-apart probe sets of *ROS1* N- and C-termini were generated by directly labeling BAC DNA (CTD-3226C9 and RP11-1059G13, respectively) with SpectrumGreen-dUTP (02N32-050, ENZO) and SpectrumRed-dUTP (02N34-050, ENZO) respectively. The NSCLC cell lines H2228 [41] and HCC78 [42] were used as positive controls for *ALK* and *ROS1* break-apart FISH test respectively. FISH assays were performed as previously described [43].

FISH signals were observed using an Olympus BX61 fluorescence microscope equipped with the appropriate filters. Enumeration of *MET* gene copy number was conducted by scoring 50 tumor nuclei; tumors with *MET* average gene copy number ≥5 were defined as positive. For *ALK* or *ROS1* break-apart analysis, the whole slide was screened and tumors meeting the following criteria were defined as *ALK* or *ROS1* rearrangement positive. 1) Broken apart: more than one set of broken apart of N-terminal and C-terminal signals exist in ≥15% tumor cells; 2) N-terminal deletion: more C-terminal signals in addition to broken apart signals in ≥15% tumor cells tumors [44].

### Dual-color immunofluorescence (IF)

PD-L1 and CD8 dual-color immunofluorescence was performed on FFPE tissue sections using rabbit anti-human PD-L1 (E1L3N) monoclonal antibody (CST#13684, Cell Signaling Technology) and monoclonal mouse anti-human CD8 antibody (IR623, DAKO). Briefly, the dual-color IF procedure was performed as follows: FFPE sections were deparaffinized and rehydrated. The antigen retrieval was then performed in a pressure cooker (PTlink module, DAKO) using high pH target retrieval solution at 97°C for 35 minutes (Target Retrieval Solution, pH 9, K8004, DAKO). Slides were then covered with a mixture of two primary antibodies and incubated at room temperature for 60 minutes, followed by two washes in TBS-T and incubation with a mixture of goat anti-rabbit Alexa Fluor 488 (A-11008, ThermoFisher Scientific) and donkey anti-mouse Alexa Fluor 555 secondary antibody (A-31570, ThermoFisher Scientific) for 30 minutes. Following two additional washes in TBS-T, slides were mounted with DAPI (H-1200, Vector). Placenta and MDA-MB-231 were used as PD-L1 positive control, whilst tonsil was used as CD8 positive control.

IF signals were observed by the same equipment as FISH. Green fluorescence staining on cell membranes represented PD-L1 positive expression, while CD8 positive expression was recognized by red fluorescent dye staining on cell membranes. Cells lacking fluorescence signals were defined as PD-L1 and/or CD8 negative.

### Statistical analysis

PD-L1 expression on tumor cells was treated as a continuous variable. The association between clinical characteristics and PD-L1 expression on tumor cells was evaluated in the whole cohort and in AD and SCC patients separately. PD-L1 expression among subgroups defined by binary clinical characteristics were compared using the Wilcoxon rank sum test. PD-L1 expression among subgroups defined by categorical clinical characteristics were compared using the Kruskal-Wallis rank sum test. If a significant difference was observed, the Wilcoxon rank sum test was used to perform pair-wise comparisons between each pair of the subgroups. PD-L1 expression among subgroups defined by ordinal clinical characteristics were compared using the Kruskal-Wallis rank sum test and the Spearman's rank correlation rho. The difference in prevalence of PD-L1 among subgroups was analyzed using the Fisher's Exact Test. No correction for multiple comparisons was performed. A two-sided *p*-value of less than 0.05 was considered statistically significant. The analyses were performed using R (version 3.1.0).

## Abbreviations

NSCLC: non-small cell lung cancer
AD: adenocarcinoma
SCC: squamous cell carcinoma
TC: tumor cells
TIL: tumor infiltrating lymphocytes
CTLA-4: cytotoxic T-lymphocyte antigen-4
PD-1: programmed cell death-1
PD-L1: programmed cell death ligand-1
PFS: progression-free survival
ORR: objective response rate
IMT-C: immune mediated therapy for cancer
IC: immune cells
IF: immunofluorescence
TKI: tyrosine kinase inhibitors
FFPE: formalin fixed and paraffin embedded
H&E: hematoxylin and eosin
IHC: Immunohistochemistry
ARMS: amplification refractory mutation system
PCR: polymerase chain reaction
FISH: Fluorescence *in situ* hybridization
